# Changes of working conditions and job-related challenges due to the SARS-CoV-2 pandemic for medical assistants in general practices in Germany: a qualitative study

**DOI:** 10.1186/s12875-022-01880-y

**Published:** 2022-11-03

**Authors:** Annegret Dreher, Viola Mambrey, Adrian Loerbroks

**Affiliations:** Institute of Occupational, Social and Environmental Medicine, Centre for Health and Society, Faculty of Medicine, University of Duesseldorf, Moorenstraße 5, 40225 Duesseldorf, Germany

**Keywords:** COVID-19, Family medicine, General practice, Germany, Medical assistant, Primary care, Qualitative study, SARS-CoV-2, Working conditions

## Abstract

**Background:**

In Germany, general practices are usually contacted first by patients with health complaints, including symptoms characteristic of SARS-CoV-2. Within general practices, medical assistants (MAs) are the first contact person for patients and perform various tasks in close physical patient contact. Working conditions of MAs have been characterized as challenging, e.g., due to low salaries, a high workload, time pressure and frequent interruptions. The potential changes of working conditions and job-related challenges experienced by MAs due to the SARS-CoV-2 pandemic have not been fully explored. We aimed to address this knowledge gap among MAs working in general practices in Germany.

**Methods:**

Semi-structured telephone interviews were conducted between March and April 2021 with 24 MAs. Medical assistants of legal age, who worked in general practices in Germany, and who were continuously employed and without change of employer in 2020 were eligible for participation. Interview recordings were transcribed verbatim and content-analyzed using MAXQDA, using deductive and inductive coding.

**Results:**

The SARS-CoV-2 pandemic posed great challenges for MAs, including a dramatic increase in workload, changes in occupational tasks, increased hygiene measures, rearrangements of work organization, childcare issues, and structural and personnel challenges within their practice. Participants described both improved but also worsened collaboration with their employers and colleagues due to the pandemic. Many MAs complained about issues regarding SARS-CoV-2-related billing processes and an increase in unpleasant patient behavior, including disregard of practice rules or frequent verbal insults. Many also did not feel adequately appreciated by politics, media, or society for their efforts during the pandemic. Positive changes were perceived to be the expansion of digital communication channels and a growing social cohesiveness of practice teams.

**Conclusions:**

Our study suggests that the SARS-CoV-2 pandemic posed great challenges for MAs. The pandemic seems to have worsened MAs’ working conditions, which had been described as challenging already prior to the pandemic. In order to improve job satisfaction and to prevent loss of healthcare personnel, measures must be taken to improve working conditions of MAs in general practices.

**Supplementary Information:**

The online version contains supplementary material available at 10.1186/s12875-022-01880-y.

## Background

Since the beginning of the SARS-CoV-2 pandemic there have been over 390,000,000 cases of infection worldwide including more than 11,750,00 cases and over 115,000 deaths in Germany (status: February 2022) [1, 2]. In Germany, general practices are typically contacted first by patients with health complaints, including respiratory symptoms characteristic of an infection with the SARS-CoV-2 virus. Besides general practitioners (GPs), medical assistants (MAs) play a crucial role in patient care in general practices. Medical assistants in Germany are among the largest occupational groups within the outpatient sector and are the first to come into contact with patients [3]. Nationwide, about 410,000 predominantly female MAs typically perform not only administrative tasks (e.g., scheduling of appointments, patient reception, ordering of consumables), but also clinical tasks that involve close patient contact, such as blood sampling, vaccinations, and standardized diagnostic procedures (e.g., ECG recordings, spirometry, or blood pressure measurement) [4, 5]. Working conditions of MAs have been reported to be characterized by low salaries, a high workload, time pressure, overtime work, frequent working interruptions and little recognition from supervising physicians [6, 7]. Accordingly, previous studies found high stress levels among MAs in Germany [6, 8]. Medical assistants with high stress levels have been shown to report poorer health [6] and worse functioning at work in terms of slips and lapses and poorer patient interaction [9]. A study by our group furthermore found MAs to report several work-related intervention needs (e.g., needs regarding working conditions and reward from their supervisor) and found these unmet needs to be associated not only with an intention to leave the employer but also to leave the MA profession [10]. As a consequence, GPs and policy makers need to pay attention to MAs’ working conditions and their unmet job-related needs to retain MAs. Special attention in this regard should be devoted to the specific working conditions and job-related challenges experienced during the current SARS-CoV-2 pandemic and in the post-pandemic period.

Considering the abovementioned adverse working conditions of MAs and possible health and occupational consequences, it is of high interest to explore to what extent the SARS-CoV-2 pandemic has affected MAs’ working conditions (e.g., in terms of the workload, occupational tasks and novel challenges). Research suggests that healthcare staff worldwide have suffered from additional strain during the SARS-CoV-2 pandemic [11–13] and thus it may be assumed that working conditions have also become more challenging for MAs. Most studies investigating the impact of the SARS-Cov-2 pandemic on the working conditions of healthcare staff have focused on physician and nursing staff [14–16]. To our knowledge, only one epidemiological study – carried out by our group – has shed light on the working conditions and job-related challenges of MAs during the pandemic. That study found very high agreement to various stressors including high levels of uncertainty about the temporal scope of the pandemic, about one’s financial situation, about contact persons for further information, and about how to act correctly [17]. The prior quantitative approach falls short, however, in fostering in-depth understanding of relevant stressors among MAs during the pandemic. Such detailed understanding can be attained through qualitative studies. The insights from such qualitative research can not only facilitate the development or implementation of measures to support MAs at work, but may also inform the preparation for future pandemics and other disruptive events that dramatically and instantly affect people’s working life. In addition, qualitative research may also reveal positive job-related experiences and procedures that have proven effective during the pandemic and that may be maintained.

## Methods

### Study aims and design

We aimed at exploring the perceived changes in working conditions and job-related challenges among MAs in German general practices during the SARS-CoV-2 pandemic. To do so, we conducted semi-structured telephone interviews using a predefined topic guide. The ethics committee of the Medical Faculty of the University of Duesseldorf approved the study (study number 2021-1370). Additional file 1 presents the completed checklist of consolidated criteria for reporting qualitative research (COREQ) [18].

### Development of the topic guide

We developed a topic guide that explored broad themes related to the research question (e.g., what changes have been experienced in terms of workload, occupational tasks and what were novel challenges). More specific follow-up questions were asked when necessary and addressed various key aspects of MAs’ professional life, i.e., workload, job control, collaboration, gratification, practice organization, resources, and supervisor behavior. These aspects had all been identified in a previous study by our group [6, 7]. The interview guide was then pretested by interviewing an independent research assistant and a full-time employed MA. Further adaptions to the interview guide were made after the first two interviews, where necessary (e.g., change of question order, slight rewording of questions for better comprehensibility). Besides SARS-CoV-2-related questions, the final interview guide also included 13 closed-ended questions to gather socio-demographic information (i.e., age, sex, educational level, country of birth of the mother, country of birth of the father), work-related information (i.e., years of working experience as MA, type of employment), practice-related characteristics (i.e., federated state of Germany, practice location, number of physicians and MAs working in practice) and SARS-CoV-2-infection information (SARS-CoV-2 cases among colleagues, own previous SARS-CoV-2 infection). The final interview guide can be found in Additional file 2.

### Participant recruitment and conduct of interviews

The Association of Medical Professions (Verband medizinischer Fachberufe e.V.) published an advertisement of the study on their webpage and social media (about 12,000 followers) on March 10th, 2021. Eligible for participation were MAs of legal age (18 and older) (for ethical and legal reasons), working in a general practice in Germany, who were continuously employed in 2020 and without any change of employer in 2020. Those latter criteria were applied to ensure that MAs were employed both before and during the pandemic and were accordingly able to compare the two periods. Employer change was an exclusion criterion to ensure that the experienced job-related changes could not be attributed to the change of employers but rather to the pandemic. Additionally, MAs were recruited via snowball-sampling to include MAs with rare characteristics (especially male MAs) in the study in order to increase the likelihood that the full range of views was captured. A detailed study information and a written consent form were then sent to MAs interested in participation via mail. They were asked to send back the signed form in order to participate in the interview. The interviews were conducted and recorded on tape by AD between March 18th and April 26th, 2021 [19]. The recordings were subsequently transcribed verbatim according to Dresing & Pehl’s simple rules [20] and anonymized by an external service provider who was bound by currently applicable regulations on data protection. Interviews were conducted in German and quotes were translated into English by a professional translator for this publication.

### Data analysis

Qualitative content analysis was performed using MAXQDA 2020 software (VERBI GmbH, Berlin, Germany) following the summarizing approach of Mayring [21]. This approach describes how during interview transcript coding, categories are formed deductively according to the research questions. Within these main categories, subcategories are formed inductively as they emerge from the research material. AD and VM independently coded five interview transcripts, compared codes and subsequently resolved discrepancies via discussion, which led to the preliminary coding scheme. AD has an educational background in epidemiology and is experienced in occupational health research [22], including research on general practices [23] and MAs [17]. VM has an educational background in public health and is also experienced in research in the field of occupational health [24] and MAs [9]. The qualitative analysis of all remaining interview transcripts was then performed by AD according to this scheme. After the first coding round, the scheme was further reviewed and slightly modified by AL, an experienced occupational health researcher with expertise related to qualitative data analysis [7, 18, 25]. This resulting coding scheme was then applied to all transcripts in a second round of coding. As only small modifications were made during the second coding round, two coding rounds were deemed sufficient.

## Results

In total, 24 interviews were conducted with an average duration of 38.4 minutes (range 21-74 minutes). No new themes seemed to emerge after 18 interviews; however, six further interviews were conducted to ascertain thematic saturation was reached. Characteristics of study participants can be found in Table 1. Participants’ mean age was 40.1 years and 95.8% were female. This sex distribution, however, reflects the overall sex distribution of the underlying population of MAs in Germany [5]. Compared to the statistics of the Federal Employment Agency (FEA), our study sample comprised more MAs working full-time (70.8% vs. 52.2%) [5]. Our sample also comprised more MAs between 25 and 54 years (91.7% vs. 65.1%) [5]. Numbers on MAs’ educational level are unavailable, however, the number of MAs with intermediate education in our sample was higher than among MA apprenticeship entrants in 2020 (79.2% vs. 55.0%) [26]. Overall, it seems that we have recruited a fairly heterogeneous group of participants, that is, from different federated states in Germany, practice sizes and practice locations as well as MAs with varying years of job experience.

**Table 1:** Socio-demographic characteristics of study participants (*n =* 24)

Characteristics	n (%)
Sex	
Male	1 (4.2)
Female	23 (95.8)
Age, mean (min-max)	40.1 (24-58)
Highest level of education	
Low^1^	0 (0.0)
Intermediate^2^	19 (79.2)
High^3^	5 (20.8)
Migrant background^4^	
Yes	3 (12.5)
No	21 (87.5)
Years in job, mean (min, max)	18.8 (1-41)
Employment status	
Full-time	17 (70.8)
Part-time	7 (29.2)
Practice location	
Urban (over 100,000 inhabitants)	12 (50.0)
Suburban (20,000 – 100,000 inhabitants)	6 (25.0)
Rural (less than 20,00 inhabitants)	6 (25.0)
Federate state^a^	
Baden-Wuerttemberg	5 (20.8)
Bavaria	4 (16.7)
Hamburg	1 (4.2)
Hesse	1 (4.2)
North Rhine-Westphalia	9 (37.5)
Rhineland-Palatinate	3 (12.5)
Saxony	1 (4.2)
Number of physicians in practice, mean (min, max)	3.4 (1-11)
Number of medical assistants in practice, mean (min, max)	7.8 (3-20)
Confirmed SARS-CoV-2 cases among practice staff	
Yes	9 (37.5)
No	15 (62.5)
Own previous infection with SARS-CoV-2	
Yes	0 (0.0)
No	24 (100.0)

^1^Secondary modern school qualification (‘Haupt−/Volksschulabschluss‘); ^2^Secondary school level 1 certificate (‘Mittlere Reife‘, ‘Realschulabschluss‘or ‘Fachschulreife‘); ^3^General qualification for university entrance (‘Abitur‘) or entrance qualification limited to universities of applied sciences (‘Fachhochschulreife‘); ^4^Migrant background defined as having at least one parent with a country of birth other than Germany; ^a^There were no participants from the nine remaining federate states of Germany

In what follows we will present changes and challenges reported by MAs during the SARS-CoV-2 pandemic grouped by three different angles, i.e., changes on the practice level, changes on the superordinate level (e.g., politics, legislation, society) and individual level changes. This grouping corresponds to a framework of macro (superordinate level), meso (practice level) and micro (individual level) level factors affecting MA’s working conditions and challenges during the pandemic. Such a framework has been largely applied in other qualitative studies [27–30] and can be seen as an adaption of the determinants of health model by Dahlgren and Whitehead [31, 32].

### Practice level

Changes on the practice level concerning e.g., workload, occupational tasks and work organization are summarized in Table 2. Whenever mentioned, the individual view of MAs was added besides the respective changes (i.e., whether they were perceived positively or negatively).

**Table 2:** Pandemic-related changes on practice level and subjective evaluation of changes by medical assistants

Domain	Changes	Evaluation of changes
**Workload**	Increase in workload (due to e.g., increased telephone traffic, E-mail traffic, performance of swab tests, administration of vaccinations, bureaucracy)	**Negative:** Unbalanced distribution of additional workload between MAs and GPs or between MAs: MAs with childcare obligations spared from additional work and new tasks mostly assigned to MAs with higher mental capacity.
	Increase in working hours (e.g., working overtime, giving up free days, working on weekends)	Not explicitly mentioned.
**Occupational tasks**	Variety of new tasks such as organizational tasks (e.g., organizing contactless transfer of receipts), tasks related to COVID swab tests (e.g., performing tests, checking testing eligibility), tasks related to COVID vaccinations (e.g., organizing and administering vaccinations, vaccinations in elderly homes and addiction clinics) and psychological tasks (e.g., calming worried patients)	**Positive:** MAs excited about new tasks and spending days outside the practice (e.g., performing tests at schools) **Negative:** Difficulties performing new tasks (e.g., counselling of patients without having relevant information, arranging vaccinations without knowing when and which vaccines will be delivered, high time expenditure for arranging vaccination appointments)
	Increased need of constantly keeping up to date with colleagues	**Negative:** Part-time working MAs felt disadvantaged in keeping up to date (compared to full-time MAs) **Negative:** Face masks hampered communication for MAs with hearing disability
**Hygiene measures**	Implementation of comprehensive hygiene measures (e.g., use of PPE, installing acrylic glass panels at registration desks, temporal relocation of the waiting area outside practice rooms, reducing waiting room seats)	**Positive:** Wearing PPE made MAs less anxious about a possible infection **Negative:** Insufficient supply with PPE and masks did not fit properly
**Work organization**	Separation of infectious patients from others (e.g., different appointment hours, separate treatment rooms)	**Negative:** Management of patients is exhausting (e.g., frequent doorbell sounds, letting in patients individually)
	Implementation of tools to manage COVID patients (e.g., checklist to ask patients about symptoms on the phone, marking potentially infectious patients in the practice system)	**Negative:** High number of aspects to be considered and kept in mind
	Reduction of patient numbers in practice (e.g., patients asked to wait outside, mandatory appointment scheduling, pre-selection of patients on the phone)	**Positive:** Appointment scheduling facilitated planning of work days **Positive:** Reduction of patient numbers brings silence to practice rooms
	Drop of high-risk medical procedures (e.g., lung function testing)	Not explicitly mentioned.
	Change in frequency of team meetings (either drop of meetings or increase in weekly meetings)	**Positive:** New team meetings kept all practice members up to date
	Stronger practice emphasis on e-mail communication, video consultations, creation of a new practice web page	**Positive:** Video consultations for elderly home residents prevent the risk of infecting vulnerable patients **Positive:** Video consultations reduce number of patients in practice rooms and provide infection protection for practice staff
**Structural and personnel factors**	Higher demand of personnel due to increase in workload	**Negative:** Difficulties attracting new staff (general practices viewed as unattractive employers during pandemic)
	Less demand of personnel due to a decrease in workload	**Negative:** Worry of MAs of becoming laid off
	Break room converted to an additional treatment room	**Negative:** No break room anymore for e.g., lunch breaks
	Practice software unable to keep up to date with frequent changes	**Negative:** MAs have to manually enter new information into the system

#### Changes in social interactions (GPs, MA colleagues, patients)

##### Changes in social interaction between MAs and GPs

In terms of changes in interaction between MAs and GPs during the pandemic, MAs reported positive and negative aspects. It was positively highlighted that GPs and MAs helped each other out during pandemic times by taking over tasks to lighten each other’s workload. Some MAs reported that GPs engaged in close discussion with them to solve any COVID-related issues. The need to find solutions jointly was perceived by MAs to lead to stronger social ties between them and GPs. By contrast, other MAs reported stressful collaboration since the start of the pandemic. This was mainly felt to be due to different expectations of GPs and MAs in handling the pandemic. For example, some MAs did not feel involved in the GPs’ decision making (e.g., when deciding to become a COVID focal practice), others explained that they showed high motivation in finding solutions for fighting the pandemic, which GPs did not appreciate (e.g., declined MAs’ ideas or scolded MAs for taking action [see Additional file 3, verbatim quote Q1]). Furthermore, MAs explained that the implementation of GPs’ instructions were not seen as feasible in practice, which led to situations when MAs at times were torn between GPs and patients (Q2):

*I personally think it’s really horrible. Because you have so much going on already, you have to think about so much, you want to make the best of everything. And then you are not supposed to discuss too much with people on the phone. At least, that’s what they’re telling us, we should not discuss with the people. But I think it sometimes makes sense to explain certain things to them, talk about how things are and why they are like that. This is then often considered as discussing. So, it’s really difficult to find the right balance; on the one hand, you don’t want to simply brush people off, you want to give them a chance of understanding everything. This is just exhausting.*

In some cases, participants expressed that they felt left alone by GPs. This ranged from situations in which GPs did not seem to care about current COVID regulations or did not want to deal with them to situations where GPs were unavailable for their practice team as they performed e.g., swab tests at local schools. The latter led to annoyance among some MAs who felt that GPs benefitted financially from the pandemic without sharing any of it with MAs. Similarly, one MA described how she felt GPs’ workload had decreased during the pandemic, whereas MAs’ workload had strongly increased leading to an imbalance of workload (Q3, Q4).

In terms of appreciation from GPs, an important aspect turned out to be financial appreciation. Some MAs highlighted that they enjoyed that they had received a COVID bonus from GPs (note: this type of tax-free bonus was a voluntary offer to MAs that GPs had to pay from their own earnings) or that the GP had adjusted MAs’ salaries to a recently introduced non-mandatory pay scale agreed to by worker unions. Others expressed disappointment as they had neither received such a bonus nor a wage adaption despite their perceived contributions during the difficult time of the pandemic.

##### Changes in social interaction between MAs and MA colleagues

Many MAs reported a closer and more intense communication with their colleagues accompanied by a decrease in gossiping and chitchat. Many also emphasized how the pandemic had strengthened their team in terms of increased appreciation for each other and increased support. This support was illustrated, for example, by the fact that MAs stood up for each other in difficult patient interactions, motivated one another, took infection control seriously to protect everyone in the team, and showed mutual understanding in special situations (e.g., regarding conflicting childcare obligations). Some MAs reported to have reduced non-urgent sick leave days and days off to support the rest of the team. Some teams had newly implemented after-work get-togethers or weekly team breakfasts.

In some cases, however, social interaction in MA teams deteriorated. Frequently, MAs reported a tense atmosphere within the team. Explanations were, among others, the high workload, the imbalance between work life and private life, stress due to a lack of personnel, frequent changes to daily workflows, highly sensitive patients, the duty to remain kind to patients, but also envy among MAs due to unequal distribution of short-time work (Temporary reduction of regular working hours due to a significant loss of work. Employers in Germany can partially compensate for a loss of wages of employees, for example, by applying for funds from the unemployment insurance through the Federal Employment Agency [33]). Some MAs did not want to be put on short-time work and envied those who were not put on short-time work. The pandemic may also have led to MAs showing less patience in day-to-day interactions with older colleagues (Q5):

*Because especially our elder colleagues, well I am not the youngest myself, but those who are around sixty years old, it’s actually too much for them, isn’t it? Because I come in every day and I tell them: You have to watch out for this. You have to remember that. This is new now. Please pass it on properly. They can’t really compensate that. Well, I really pity them, but I have to make sure they can recall it. And this is the worst. I can’t be considerate of them because they don’t know it. They have to know it just like an 18-year old employee here.*

##### Changes in social interaction between MAs and patients

Participants mentioned that hugging patients (when e.g., their spouse had died) was no longer possible during the pandemic and that they missed this. Face masks were perceived to hamper communication as facial expressions could not be noticed and consequently created a feeling of distance between them and their patients. The strict hygiene measures were reported to lead to a reduction of short private conversations about patients’ families and private matters. Medical assistants reported to miss these conversations which had otherwise been part of their daily work.

Besides social interaction, professional interaction with patients has also been reported to have changed during the pandemic. One MA explained that she regularly had to limit the duration of phone calls due to time restrictions and that this made her cut short on the phone, which she regretted. Another MA stated that there were days where she struggled to remain friendly on the phone.

Overall, many MAs reported an increase in long and exhausting discussions with patients since the onset of the pandemic. The most frequent topics of discussion were, according to interviewees, the prioritization in the administration of COVID vaccinations, patients’ preferences for certain vaccines, the lack of vaccines for all patients, and vaccine safety. Patients were viewed to regularly ask for vaccination prioritization by elaborating on pre-existing conditions and ignoring the priority order of vaccinations set by the practice. In other cases, MAs felt they had to justify why there were not enough vaccines available and why patients had to wait for their turn. One MA described that there had never been such a need to discuss issues related to vaccine safety with patients before and that she frequently had to argue with patients now (Q6):

*So, everyone in the practice got [vaccine brand]. I am glad if I receive a vaccine at all, I also tell that to anyone starting like: “Well yes, but I don’t want that one”, I tell them: “Be glad if you receive anything at all.” Because nobody has ever asked me about the vaccinations against yellow fever, typhus or malaria before their vacation to Africa, nobody has asked me how long these vaccines have been researched, which side effects they have. Never ever.*

#### Patient-related changes

##### Changes in patient numbers

Many MAs reported a strong decline in patient numbers, especially at the beginning of the pandemic. According to participants, patients preferred calling the practice instead of showing up in person due to a fear of infection with SARS-CoV-2. Some MAs reported that regular check-ups were also cancelled due to patients’ fear of becoming infected and that patients did not show up even in cases of emergencies (Q7). Partially, this decline in patient numbers was felt to persist until the conduct of the interviews. Some MAs viewed the decrease in patient numbers positively as it led to a calmer work routine. Others, in contrast, found the emptiness of practice rooms frightening as they were worried about patients not showing up although they needed treatment. However, other MAs reported that in the further course of the pandemic the trend of declining patient numbers changed rapidly as soon as flu vaccinations and COVID vaccinations were available and recommended by official authorities. One MA stated that she had never experienced such a strong demand by patients to receive flu shots in her entire professional life. This patient rush led to stress and a feeling of overload as GP practices usually order flu vaccines in the previous year and were not prepared for this high demand for vaccines. Medical assistants furthermore described that they perceived an immediate impact of media reporting on patient numbers (e.g., huge patient rush the day after the official announcement of the uptake of swab tests or vaccinations by general practices) (Q8).

Medical assistants working in designated COVID focal practices reported an increase in patient numbers as other general practices refused to treat patients with flu-like symptoms. These patients were then referred to the focal practices that were thus overloaded. One MA explained an additional reason for high patient numbers: in times of the pandemic, employers more frequently sent home employees with flu-like symptoms who in turn had to show up at general practices to receive a sick leave certificate. Some MAs reported that the type of patients visiting their practice had also changed (e.g., increase in consultations regarding mental health, increase in patients with flu-like symptoms, increase in foreign English-speaking patients who traveled and needed a COVID test certificate).

##### Patients’ anxiety and behavior

A theme commonly expressed by participating MAs pertained to changes of patients’ attitudes and behavior during the course of the pandemic. Several MAs described that they felt that patients had high expectations of MAs during the pandemic. In their eyes, MAs were, among others, not only expected to solve problems immediately, but also to be constantly up to date with any current COVID regulations, to promptly reply to patients’ requests, or to instantly provide swab test results. Some MAs stated that they felt that patients viewed them as robots that were not allowed to make mistakes. Participants often described feeling as if their work was not seen (Q9):

*It was really frustrating for me, […] they always think that when consultation hours begin / well that the girls [note: MAs] are there, period. They don’t know that we take care of the urgent ones at noon, we do the paperwork and then the afternoon begins. 12-hour-shifts are not unlikely to happen, it’s pretty easy. And they don’t see that, do they?*

According to most MAs’ reports there was large variation of the changes in patient behavior: some patients were described to express increased gratitude and appreciation for MAs in the form of gifts (e.g., food and sweets) and kind words, whereas other patients seemed to become more demanding, aggressive, and selfish. Behavior was also described to vary with patient age: for instance, younger patients often demanded information from MAs despite being able to obtain the needed information by themselves whereas older patients were often unable to obtain information, but were not asking for it (Q10). Patients were frequently experienced as showing no understanding of COVID regulations, the reasons for not getting through on the phone, the lack of swab test capacities, longer waiting times, and new practice organization approaches (e.g., prescription collection restricted to certain times of the day, patients with flu-like symptoms restricted to certain consultation hours). One MA described how patients even made claims on national holidays (Q11).

Participants illustrated how patients did not adhere with COVID regulations and practice organization (e.g., trying to enter practice rooms through the windows when the door was still closed, ignoring information signs or wearing face masks inappropriately). Patients were described as being confused by media reports, insecure due to the ever-changing governmental action, and overwhelmed by the pandemic itself. This led to partially irritated and unpleasant behavior. Patients were reported to frequently verbally abuse MAs on the phone or were perceived to behave aggressively e.g., when test results were not provided fast enough or when patients did not receive an appointment for vaccination. Further reasons of patients’ negative emotions were reported to be the poor reachability of practice staff by phone and the occasional inability of MAs to provide reliable information. Other patients seemingly expressed their discontent in the form of negative online reviews for the practice. These changes in patient behavior, as described by MAs, often resulted in psychological distress for them. Some explained how they felt exhausted, frustrated, personally attacked, at the mercy of patients or that they often cried after being insulted. Two MAs described that the excessive patient demands made them question their jobs.

## Superordinate level (politics, legislation, society)

### Information flow

As reported by MAs, a major challenge during the pandemic was obtaining information. Some MAs complained about a poor information flow on the part of the government to general practices. An example mentioned were patients who had received letters on the necessity of testing clear after quarantine from the government while MAs had not received this information and neither had their contact person at the local health authority. Others claimed their regular contact partner, the Association of Statutory Health Insurance Physicians, was difficult to reach and if reached, contact persons could not always provide the requested information. Many MAs described an information imbalance between them and other parties. It appeared, for example, to MAs as if certain general practices had more information than others. Furthermore, some MAs explained how the press seemed to have more information than general practices (Q12). Frequently, MAs also described situations in which patients had more information than they had. Finally, MAs expressed their dissatisfaction about different information flow across federated states (Q13) and contradicting information from the government. Examples of missing information were e.g., information on administration and billing of COVID services, COVID vaccination procedures and planning, or the procedure of referring patients to vaccination centers (Q14, Q15):

*So, eventually it was said that there were corona tests once a week for everyone in [location]. Of course, we do it because we also want to help. We didn’t know how to do the billing, we didn’t know what to do, we didn’t know if we had to type a diagnosis or not. We had to get all the information by ourselves. And I was also sitting there, talking on the phone, doing research on the internet, gathering all the information. So, there was nothing else, nobody told us, this is how you have to do it.*

### Media reporting

The main issues regarding media coverage during the pandemic as perceived by MAs were the invisibility of MAs in general in pandemic-related media reports (Q16) and the discrepancy between media reports and everyday work life in general practices (Q17):

*It’s presented differently in the media. Because everyday life looks different, it’s really sugarcoated on television, isn’t it? Not everyone is tested for the mutation, for example. They are only tested if there is concrete evidence or rather if we have a suspicion, like the course of disease has been pretty quick, let’s also test for the mutation.*

The latter was presented as one reason for increased discussions with patients who made claims based on media reports (e.g., demanding immediate vaccination appointments after the media had announced that general practices would offer vaccinations).

### Politics and MAs

The action of policy makers during the pandemic was experienced in different ways by participants. Some MAs expressed satisfaction with governmental support during the pandemic. For instance, they appreciated being equipped with personal protective equipment (PPE) by the Association of Statutory Health Insurance Physicians and liked the content and form of received information brochures. Nevertheless, other MAs complained about a lack of governmental support. This was partly because they were unhappy about the PPE that had been sent to them as it was deemed insufficient in terms of both quantity and quality. Furthermore, reimbursement for COVID services was viewed as too low. A further aspect was the perceived invisibility of MAs in politics: participants explained how politicians never seemed to talk about them (Q18) and did not offer any financial bonuses for MAs whereas other medical professions had received them [note: the federal government of Germany covered a financial bonus in 2020 for certain occupational groups e.g., geriatric nurses [34]. For MAs, however, no such federal bonus existed and only an optional bonus could be paid directly by GPs].

Medical assistants felt that they were not considered when new policies were implemented during the pandemic. Examples include when policy makers determined which occupational groups were eligible for early COVID vaccination. Medical assistants were initially not a priority group and MAs thus complained they had received their offer for vaccination way too late (Q19). The same applied for the right to be regularly tested for SARS-CoV-2 free of charge.

### Bureaucratic and legal changes

A core theme that regularly emerged from interviews were bureaucratic and legal changes during the pandemic. The implementation of policies, for example, was found to be problematic: Participants mainly described how the implementation of new regulations did not seem to be feasible in practice, were on short notice, seemed to be futile, and were complicated by considerable amounts of bureaucracy (Q20, Q21). For example, in the process of COVID vaccination, the ordering process of vaccines was deemed too complicated, and politicians publicly promoted vaccination despite a lack of vaccine supplies in practices.

Participants also complained about an unprecedented accumulation of changes and explained how they lacked lead time for implementation (Q22). In some cases, even retrospective changes were reported that required a subsequent adjustment of e.g., billing numbers that had already been applied (Q23). Major bureaucratic changes reported by MAs concerned the additional steps needed when treating COVID patients (e.g., new forms, new entry options into their practice system, many and complex billing numbers), changes in SARS-CoV-2 vaccination and testing entitlement, or changes in influenza vaccination recommendations (Q24). The majority of participants agreed that particularly constant changes in billing numbers made their everyday work life difficult (Q25).

*In the fourth quarter, there was a whole table on how to do the billing. This overview has changed three times in one quarter. […] So we simply/ We couldn’t keep track of it. I had three different sheets with information on how to do the billing in the different periods. And it was basically in the same quarter. It was 3 months.*

One MA explained how she had never experienced such an amount of bureaucracy in 35 professional years. Others described how bureaucracy took precedence over patient care (Q26, Q27).

## Individual level

### Emotional and psychophysiological reactions

Participants reported that the pandemic had caused a wide range of emotions and psychophysiological reactions. These ranged from the fear of becoming infected with the virus (Q28), uncertainty about the infection risk posed by the pandemic, and annoyance by the omnipresence of the pandemic in their life (i.e., the pandemic was present at work and continued to be present at home in media and private conversations). Some reported feeling additionally anxious about infection after relatives had died from the virus. Participants explained how either they themselves or colleagues suffered from cardiac arrhythmia, migraine attacks, or fatigue which were attributed to the pandemic. Others referred to sleep disturbances and not being able to mentally withdraw from work at home. In contrast, some MAs said that they had accepted the pandemic situation, which led to calmness. Again, others held positive views, e.g., of the pandemic as a challenge as they claimed to feel the need to help others and were glad about the opportunity to contribute.

### Work-family conflict

According to MAs, their job also widely impacted their private life during the pandemic. Medical assistants reported that friends and family kept more physical distance from them and their children out of fear of infection (Q29). In turn, some MAs were particularly worried about infecting family members due to the high infection risk their job posed. Regarding working hours, several participants described how they continued working in their free time by collecting information and preparing their next working day (Q30). In some cases, MAs’ private life was also described to influence their profession, for example, when MAs needed to leave work at fixed hours to pick up their children from childcare. Childcare overall posed challenges for MAs because, despite entitlement to childcare due to their health care profession, daycare centers were reported to cut hours and MAs faced high levels of bureaucracy to be able to use childcare services (Q31, Q32).

### Job satisfaction and change of job

Some MAs reported no change in job satisfaction. One MA explained she was convinced that changes were part of MAs’ jobs and must always be expected. Others explained how job satisfaction was high before the pandemic, temporarily decreased due to e.g. high patient demands in times of vaccine introduction, but quickly returned to pre-pandemic levels. Few expressed an increase in satisfaction due to new job tasks emerging from the pandemic situation and being given new areas of responsibility with which they identified. However, most MAs described that their job satisfaction had decreased in the long term and that they questioned the meaningfulness of their profession more frequently due to either an increasing numbers of patient insults, a lack of societal appreciation and visibility, a lack of governmental support, or misleading media communication during the pandemic (Q33). In line with this, some MAs explained how they had either recently quit their job or considered quitting. Some considered a change of specialty.

## Possible interventions expressed by MAs

During the interviews, many MAs expressed possible interventions to different addressees during the pandemic. These covered a wide range of topics and highlight which points were considered particularly important to MAs. A summary of possible interventions can be found in Table 3.

**Table 3:** Interventions expressed by *n =* 24 MAs during the COVID pandemic

Addressee	Intervention	Examples
To the media	More appreciation of MAs	• Mentioning MAs in media reports
	Presentation of MAs’ working day in media reports	• Public education about the real situation in practices
To politicians	More appreciation of MAs	• Considering MAs eligible for early vaccination • Expressing appreciation for MAs
	Listening to the needs of MAs	• Replying to letters sent by MAs’ professional association
	Financial support during pandemic	• Government paying a COVID bonus
	Protection of MAs during pandemic	• Outsourcing of COVID services to designated corona centers
	Simplified access to childcare for system-relevant professions	• Reduction of bureaucracy when applying for childcare
	Action after consultation with and in accordance with practices	• Exploring views and needs in practices before decision-making • Informing practices about innovations/adaptations before informing the public • Direct contact with practices, not via media • Bundling of information on changes, not new ones every day • More lead time before implementation of changes • Better supply with PPE** • No advertising of vaccination if supply is insufficient • Reduction of ICD codes for billing
	Clear guidelines of action	• Clearly defined order of COVID vaccination eligibility
To GPs	More appreciation of MAs	• Expressing appreciation to MAs • Paying a COVID bonus
	Support of MAs during pandemic	• Active collaboration on joint problem solutions
	Financial participation	• Sharing additional revenue from pandemic with staff
To patients	More appreciation of MAs	• Expressing appreciation to MAs, e.g., verbally or with gifts
	Better patient behavior	• Better compliance with practice rules and acceptance of procedures • Taking pandemic more seriously • Acting friendlier and less selfish
No special addressee	More physical proximity to patients	• Hugging patients, shaking hands
	Alleviation of work organizations	• Adopting weekly team meetings • Keeping PPE** in stock for the future • Normalization of working hours

*MAs Medical assistants, **PPE Personal protective equipment

## Discussion

This is the first qualitative study to explore changes and challenges related to the working life of MAs due to the SARS-CoV-2 pandemic. Our findings suggest a major impact of the pandemic on MAs’ daily work, including a significant increase in workload, bureaucracy, stress due to inadequate government communication, unpleasant patient behavior, a general perceived lack of appreciation of MAs, but also growing social cohesiveness of practice teams. Medical assistants explicitly expressed possible interventions to GPs, patients, to the media and to politicians that included, among others, a desire for more appreciation, stronger involvement in pandemic procedures, governmental support (both financially and in terms of PPE and clear guidelines), better governmental communication and better patient behavior.

Several international studies have previously investigated the situation of GPs during the pandemic and report findings similar to those observed in this study, e.g., an increase in workload [35, 36], increased hygiene measures [35, 37], worry of becoming infected [36, 38], adoption of remote patient consultations [37, 39], a lack of PPE [35, 37, 39, 40], unfavorable media broadcasting [41], difficulties in obtaining relevant information [35], and difficulties in handling the amount of information by health authorities [36, 42]. However, those studies solely represent GPs’ views and failed to address challenges specific to MAs. Challenges during the pandemic reported by the MAs in our study that were not mentioned in previous research on GPs were, for instance, the sharp increase in unpleasant patient encounters, frequent changes in billing processes that required adaptations, a general lack of appreciation for the work being done during the pandemic, challenging teamwork with GPs and colleagues, the unmet desire for a financial bonus, and childcare issues. Regarding the increase in workload due to the pandemic, MAs felt that great parts of the workload rested primarily on their shoulders instead of GPs’. Most of the current evidence from general practices during the pandemic [36, 37, 40–42] furthermore builds on quantitative methodologies or qualitative explorations of very specific topics (e.g., remote patient consultations) and may therefore not be able to facilitate an understanding of the full scope of the challenges encountered by general practice teams during the pandemic. Thus, to our knowledge, our study is the first to provide detailed insights into the experience of everyday working lives in general practices according to MAs, and to describe in greater depth why certain challenges were perceived as such (e.g., PPE issues due to poor quality of material and bad fit, increase in workload due to increased telephone consultations and psychological counselling of patients).

Certain observations in this study are consistent with findings from our quantitative survey of MAs in April 2021 [17] (e.g., MAs’ increased workload during the pandemic, feeing burdened by thoughts of a possible infection with SARS-CoV-2, feeling burdened by one’s childcare situation, not feeling adequately informed by the employer). However, the findings of the present qualitative study allow for in-depth understanding of the quantitatively assessed stressors. During interviews, for example, MAs clarified which tasks led to an increase in workload (i.e., telephone consultations, swab tests, vaccinations, and hygiene measures) and why these tasks were necessary (i.e., insecurity of patients, lack of clear information flow). Regarding childcare, MAs explained how bureaucratic challenges made childcare difficult. With respect to not feeling adequately informed by the employer, MAs added that they would have wished for better preparation (i.e. clear action guidelines) and support (i.e. PPE provision) by politics rather than their direct employer. In addition to these examples, there were also various themes that emerged from interviews that were not part of the quantitative inquiry such as social interactions between MAs and other MAs, GPs and patients. Especially the latter was perceived as burdensome and one of the major stressor of MAs during the pandemic which the quantitative approach failed to capture.

The present study suggests that the pandemic has posed great challenges for MAs in their everyday work life. When interpreting the results, it must be kept in mind, however, that working conditions and stress levels of MAs have been found to be precarious even before the pandemic [8, 43, 44]. In terms of work stress according to the effort-reward-imbalance model by Siegrist [45] a previous study by our group found that nearly three quarters of MAs in Germany suffered from work stress before the pandemic [6]. Work stress according to Siegrist is defined as high work efforts (e.g., high workload) paralleled by low rewards (e.g., salary, recognition, support) [45]. This is of special interest in times of the pandemic as it appears as if efforts for MAs have additionally increased whereas rewards have not adequately increased (e.g., lack of financial compensation and appreciation by politics and society). The need to improve working conditions for MAs in general practices, especially now in times of the pandemic, is supported by a previous study from our group: We found lacking appreciation by the supervisor – a major issue according to MAs – to be amongst the strongest determinants of intentions to leave the employer or the profession among MAs [10]. A different cross-sectional study among MAs in Germany also reported social support as a protective factor for the mental workload among MAs [43]. Another study from our group referred to positive patient interaction as a main reason for MAs to remain in their profession despite high workloads [7]. The present study suggests that patient interactions have also deteriorated in the course of the pandemic, potentially contributing to more MAs ultimately leaving their profession.

### Strengths and limitations

The present study addresses changes and challenges during the pandemic specific to MAs and challenges that differ from those of GPs and that have received little attention in research. A strength of the present study is the very broad range of characteristics of the included participants regarding their age, migrant background, working experience, employment status, practice location (urban, suburban, rural), federated states, and practice size (number of MAs and physicians), which likely maximizes the likelihood that we included participants with a broad range of views. Thematic saturation was felt to be reached after 18 interviews and even after conducting six more interviews, no new themes emerged, which supported the assumption that thematic saturation had been reached. Regarding this study’s limitations, a first limitation is the possible selection bias in recruiting participants. Possibly, MAs who felt especially burdened by the pandemic were more likely to participate. However, some participants also reported that there had been only few changes in their work life and many positive changes. A further limitation is that no participants with low educational level and no MAs in training were recruited for this study. We cannot rule out that those groups would have reported different changes and perceptions during the pandemic. Official numbers of the federal employment agency suggest that 22% of MAs have a low educational background [46]. In other studies from our group, however, the numbers of MAs with low educational background were low, too [17, 25]. This may suggest a decreased interest in study participation among this subpopulation. Although it remains unclear if and how study results were affected, a possible limitation may be that our sample comprised more MAs in the age group of 25-54 years compared to the underlying population of MAs in Germany. Despite the reach of the Association of Medical Professions, it remains unclear how many MAs were reached by the study advertisement. Some further limitations concern the applied methodology. Although qualitative content analysis is a well-suited approach for a systematic analysis of large quantities of data, it may be possible that individual quotes and opinions lose meaning during the formation of categories when reducing the data material [47]. Only one coder performed the full qualitative analysis. However, a second independent coder was involved in the formation of the coding scheme, and the final scheme was reviewed by a third independent researcher who approved the scheme after few adaptations. Nevertheless, all included researchers share a similar academic background and it would probably have enriched the analysis to include a primary care researcher or MAs. Finally, as it is typical in qualitative research, personal attitudes and experiences of the coders and data analysts may have influenced analyses.

## Conclusion

The present study suggests that the SARS-CoV-2 pandemic posed great challenges for MAs in general practices. Stressors specific to MAs that have not been reported in research among GPs were, among others, unpleasant patient encounters, issues regarding billing processes, a lack of appreciation for the pandemic-related effort, the desire for a financial bonus, and childcare issues. However, also positive pandemic-related changes were mentioned, such as the expansion of digital communication channels and a growing social cohesiveness of practice teams. Nevertheless, it appears as if the pandemic has aggravated pre-existing issues regarding MAs’ working conditions (e.g., long working hours, low salary, and low appreciation). Possible starting points for interventions to improve MAs’ working conditions may be informed by MAs’ intervention suggestions to GPs, patients, to the media and to politicians. These included, among others, a desire for more expressed appreciation, compassion and understanding on the part of patients and GPs, stronger involvement of general practice staff in pandemic procedures, governmental support (e.g., a. COVID bonus as a financial recognition, adequate PPE supply, reduced bureaucracy, taking MAs into account for early vaccination eligibility, and clear action guidelines), and better governmental communication. Our results call for future quantitative studies to quantify the exact burden of challenges specific to MAs in general practices during the SARS-CoV-2 pandemic to improve their working conditions in the long term.

## Supplementary Information

**Additional file 1.** Completed checklist of consolidated criteria for reporting qualitative research (COREQ).

**Additional file 2.** Interview guide for phone interviews with medical assistants in Germany about the SARS-CoV-2 pandemic.

**Additional file 3.** Verbatim quotes of MAs.

## Data Availability

Data cannot be shared publicly because the transcripts contain highly sensitive information (e.g. own mental stress, conflicts with employers and colleagues, revelation about hygienic procedures during pandemic). The ethics committee of the medical faculty of Düsseldorf would like to share the data on request only. Requests to access the data can be sent to Ethikkommission@med.uni-duesseldorf.de. The category system of qualitative content analysis is available from the corresponding author on reasonable request.

## Abbreviations

COREQ: Consolidated criteria for reporting qualitative research
COVID: Coronavirus disease
ECG: Electrocardiogram
GP: General practitioner
ICD: International classification of diseases
MA: Medical assistant
PPE: Personal protective equipment
SARS-CoV-2: Severe acute respiratory syndrome coronavirus type 2
