# Supplementary material for: Changes of working conditions and job-related challenges due to the SARS-CoV-2 pandemic for medical assistants in general practices in Germany: a qualitative study
Source: BMC Prim Care. 2022 Nov 3;23:273. doi: 10.1186/s12875-022-01880-y (PMC9632591; doi:10.1186/s12875-022-01880-y)
Supplement: Supplementary file 1 — Additional file 1. Completed checklist of consolidated criteria for reporting qualitative research (COREQ). [file 12875_2022_1880_MOESM1_ESM.pdf]

Supplementary Material

Consolidated criteria for reporting qualitative research (COREQ; [1])

No	Item	Description
**Domain 1: Research team and reflexivity**		
Personal characteristics		
1.	Interviewer	Annegret Dreher
2.	Credentials	Annegret Dreher (M.Sc.), Viola Mambrey (M.Sc.), Prof. Dr. Adrian Loerbroks
3.	Occupation	AD,VM: research associate; AL: university professor and working group leader
4.	Gender	AD,VM: female; AL: male
5.	Experience and training	AD: educational background in epidemiology, practical experience in occupational health research<br>VM: educational background in public health, practical experience in occupational health research<br>AL: educational background in epidemiology and public health, practical experience in occupational health research, qualitative and quantitative research and teaching
Relationship with participants		
6.	Relationship established	No
7.	Participant knowledge of interviewer	Participants knew AD as a researcher in the field of medical assistant health from prior publications and advertisement of the study
8.	Interviewer characteristics	No other characteristics were reported about the interviewer
**Domain 2: Study design**		
Theoretical framework		
9.	Methodological orientation and theory	Qualitative content analysis by Mayring
Participant selection		
10.	Sampling	Convenience and snowball sampling
11.	Method of approach	Online distribution of the study call
12.	Sample size	24 participants
13.	Non-participation	Not applicable
Setting		
14.	Setting of data collection	Participants participated in telephone interviews at a location of their choice
15.	Presence of non-participants	No
16.	Description of sample	Between 24 and 58 years (M = 40.1), 23 females, 1 male, MAs from entire Germany
Data collection		
17.	Interview guide	Provided as supplemental material; Interview guide was piloted before study
18.	Repeat interviews	None
19.	Audio/visual recording	Audio recording
20.	Field notes	None
21.	Duration	21 – 74 minutes (M=38.4)
22.	Data saturation	Yes
23.	Transcripts returned	No
**Domain 3: Analysis and findings**		
24.	Number of data coders	Two (AD, VM)
25.	Description of coding tree	Yes (in the results section)
26.	Derivation of themes	Deductive coding: changes in everyday working life of MAs, perception of changes, barriers and enablers; all other categories were inductively coded
27.	Software	MAXQDA 2020
28.	Participant checking	No
Reporting		
29.	Quotations presented	Yes
30.	Data and findings consistent	Yes
31.	Clarity of major themes	Yes
32.	Clarity of minor themes	Yes

1. Tong A, Sainsbury P, Craig J. Consolidated criteria for reporting qualitative research (COREQ): a 32-item checklist for interviews and focus groups. International Journal for Quality in Health Care. 2007;19(6):349-357.
