# Supplementary material for: Changes of working conditions and job-related challenges due to the SARS-CoV-2 pandemic for medical assistants in general practices in Germany: a qualitative study
Source: BMC Prim Care. 2022 Nov 3;23:273. doi: 10.1186/s12875-022-01880-y (PMC9632591; doi:10.1186/s12875-022-01880-y)
Supplement: Supplementary file 2 — Additional file 2. Interview guide for phone interviews with medical assistants in Germany about the SARS-CoV-2 pandemic. [file 12875_2022_1880_MOESM2_ESM.pdf]

Changes of Working Conditions and job-related Challenges due to the SARS-CoV-2 Pandemic for Medical Assistants in General Practices in Germany: A Qualitative Study

A study conducted by the Institute of Occupational, Social and Environmental Medicine, University of Duesseldorf

institut arbeitsmedizin sozialmedizin & umweltmedizin

Interview Guide

1. Welcoming

Good afternoon and welcome. First of all, I would like to introduce myself: My name is Annegret Dreher and I am a research associate at the Institute of Occupational, Social and Environmental Medicine at the University of Duesseldorf.

2. Explain the aim of the survey

This interview is part of my doctoral thesis. I am investigating the impact of the Corona pandemic on medical assistants in Germany.

In our conversation today, I would like to learn more about how the Corona pandemic has affected and changed your personal everyday working life. I would also like to talk to you about how you personally experienced these changes. At this point, it is important to say that there are no right and wrong answers. You are completely free to reflect your personal experiences and assessments. The interview itself will last approximately 20 to 45 minutes.

3. Confidentiality and anonymization of data

All data collected will be handled in accordance with current data protection regulations. The conversation will be recorded on tape. I will NOT address you by name during the interview so that you remain anonymous. The tape recording will be typed up afterwards by an external service provider and then destroyed immediately. Any information you provide about places or names (e.g. from your employer) will NOT be typed up and will therefore not be included in the data analysis. The external service provider is subject to a confidentiality agreement.

You can terminate the interview at any time without giving reasons. This will not result in any disadvantages for you.

4. Check whether consent form is available

I have received your consent form.

5. Open questions

Before we start the interview, are there any open questions from your side?

6. Switch on tape!

I will now turn on the tape and record a code at the beginning. This code will help us later to assign the interview.

Eligibility criteria	
To get started, I'd like to ask you 3 quick questions about your employment:	
**Were you continuously employed as a medical assistant in 2020?**	□ Yes<br>□ No (e.g. unemployment, maternity leave)
**Have you changed employers in 2020?**	□ Yes<br>□ No
**Was your employer a general practice in 2020?**	□ Yes<br>□ No, other: ____________________

Interview questions	
Great, then we can continue with the content-related questions!	
**Everyday work during pandemic**	At first I would like to know…..<br>□ What does a typical working day currently look like for you?<br>▪ What tasks do you currently typically perform at your job?
**Changes**	You stated earlier that you were continuously employed as an MA in 2020. This means you also experienced the time BEFORE the Corona pandemic. What would you say....<br>□ What changes have there been at your work since the Corona pandemic began?<br>▪ How has your workload changed?<br>- Do you have more work to do?<br>- What has become more? What has become less?<br>▪ To what extent have work processes changed?<br>- Opening hours changed?<br>- Consultation hours changed?<br>- Hygiene measures introduced?<br>- Documentation changed?<br>- Range of services changed?<br>▪ Have there been any SARS-CoV-2 cases among your practice team?<br>▪ To what extent has the collaboration changed?<br>- with your boss<br>- with your colleagues<br>- with patients<br>▪ To what extent has your enjoyment of the job changed?<br>▪ To what extent has the appreciation towards you changed?<br>- by society

	- by patients<br>- by your boss<br>□ (name an example for a change) How have you personally experienced this change?<br>▪ What impact did this change have on you?<br>▪ How did you feel about it?
**Enablers**	□ (If there was anything) What helped you most during this time?<br>(Examples: What role did the team play, behavior of patients, what role did your individual tasks play?)
**Barriers**	□ (If there was anything) What made the situation particularly difficult for you?<br>(Examples: What role did the team play, behavior of patients, what role did your individual tasks play?)
**Further evaluation of changes**	□ Assuming Corona would no longer be an issue tomorrow, which of the abovementioned changes would you like to keep?<br>(Give examples for changes from above if necessary!)<br><br>□ Why would you keep these changes?

7. End of the interview

This is all from my side now. We've talked about a lot of things, but there may be things that haven't been mentioned yet. Is there anything from your side that hasn't come up yet but is important to you?

To conclude the interview, I would like to ask you a few questions about yourself:

We want to make sure that we include as diverse participants as possible in our study (young/old, full-time/part-time, big city/rural, etc.)

**Sex**	□ male<br>□ female<br>□ non-binary
**Year of birth**	_ _ _ _ (year 4 digits)
**What is your highest level of education?**	□ Finished school without graduation<br>□ Secondary modern school qualification (‘Haupt- oder Volksschulabschluss‘)<br>□ Secondary modern school level 1 certificate (‘Realschulabschluss / Mittlere Reife /Fachschulreife‘)<br>□ General qualification for university entrance or entrance qualification limited to universities of applied sciences (‘Abitur/Fachhochschulreife‘)<br>□ Other (e.g. acquired abroad)
**In which country was your mother born?**	□ Germany<br>□ Other
**In which country was your father born?**	□ Germany<br>□ Other
**Since when have you been working as a medical assistant?**	_ _ _ _ (year 4 digits)
**To what scope do you currently work as a medical assistant?**	□ Full-time<br>□ Part-time
**The practice in which you work is located...**	□ in an urban area (More than 100.000 inhabitants)<br>□ in a suburban area (20.000 to 100.000 inhabitants)<br>□ in a rural area (Less than 20.000 inhabitants)
**The practice where you work is located in....**	□ Baden-Württemberg<br>□ Bavaria<br>□ Berlin<br>□ Brandenburg<br>□ Bremen<br>□ Hamburg<br>□ Hesse<br>□ Mecklenburg Western Pomerania<br>□ Lower Saxony<br>□ Northrhine-Westphalia

	□ Rhineland Palatinate<br>□ Saarland<br>□ Saxony<br>□ Saxony-Anhalt<br>□ Schleswig-Holstein<br>□ Thuringia
**Number of physicians in the practice**	_ _ (number, 2 digits)
**Number of MAs in the practice (including yourself)**	_ _ (number, 2 digits)
**Have there been any confirmed cases of SARS-CoV-2 on your practice team?**	□ Yes<br>□ No
**Have you already tested positive for SARS-CoV-2 yourself?**	□ Yes<br>□ No

8. End of the interview

I would like to thank you very much for the helpful interview!

I will now stop the tape recording and end the interview.
