# Supplementary material for: Changes of working conditions and job-related challenges due to the SARS-CoV-2 pandemic for medical assistants in general practices in Germany: a qualitative study
Source: BMC Prim Care. 2022 Nov 3;23:273. doi: 10.1186/s12875-022-01880-y (PMC9632591; doi:10.1186/s12875-022-01880-y)
Supplement: Supplementary file 3 — Additional file 3. Verbatim quotes of MAs. [file 12875_2022_1880_MOESM3_ESM.pdf]

Topic		Verbatim quotes	Participant
**Practice level**			
Changes in social interaction between MAs and GPs	Q1	So, I am the organizational talent in our practice, this is also what I'm known for, which is why I have prepared all of the COVID vaccinations. So, my GPs, one of them just had to drive out and the other one, who was on-site, was vaccinating in the practice. And it was some real tinkering. And if you get to hear: "Well, how can you just pass on appointments? We don't even have any time slots yet." Right? But we have to monitor […] that the people come back for another vaccination after three or four weeks, or sometimes after twelve weeks. And I have to say, I really didn't like that, I was in fact very upset, because I just wanted to help. But yesterday, I was really offended when being scolded for that.	ID 4205<br>Female<br>44 years<br>28 years experience
	Q2	I personally think it's really horrible. Because you have so much going on already, you have to think about so much, you want to make the best of everything. And then you are not supposed to discuss too much with people on the phone. At least, that's what they're telling us, we should not discuss with the people. But I think it sometimes makes sense to explain certain things to them, talk about how things are and why they are like that. This is then often considered as discussing. So, it's really difficult to find the right balance; on the one hand, you don't want to simply brush people off, you want to give them a chance of understanding everything. This is just exhausting.	ID 3229<br>Female<br>27 years<br>4 years experience
	Q3	I would say the GPs have less work to do. Like, all this bureaucracy, whenever a note arrives, how do you have to bill that, they simply don't care, because they don't usually do the billing either. They say: "It's your task, see how you deal with it". And then there are only suggestions that can't be implemented. Well, technically, we [note: MAs] have re-arranged ourselves during the pandemic, we came up with solutions, we found out how things should be done. For them [note: GPs], the only real change is working with face masks, wearing protective clothing and doing swab tests from time to time. But besides that, there are no real changes for them, I think. And because there is more time between appointments, so that the waiting room is not too crowded, they also have some time in between to drink a coffee, don't they?	ID 2199<br>Female<br>47 years<br>30 years experience
	Q4	If we have, let's say, a hundred percent of additional work because of the pandemic, at least 80% of it is upon the assistant and 20% gets to the GP. Because it' usually the GP who sees the patients, if they have a cold now, they would have also gone seeing him. He would have examined them, written a prescription, given them an incapacity certificate. Right now, he is basically doing the same, the only difference for	ID 2199<br>Female<br>47 years

		him is that he has to make some decisions; is it a patient with suspected COVID infection, is a smear test necessary, he puts on protective clothing and so on, but besides that, there is not much of a difference for him. I actually think that GPs have to work less, because it is us who answer all the questions, we stretch appointments to avoid having too many patients in the waiting room. So, if you look at the extent of our work at the registration, there is only a small part that gets to the GP's room. We take care of most things, don't we?	30 years experience
Changes in social interaction between MAs and MA colleagues	Q5	Because especially our elder colleagues, well I am not the youngest myself, but those who are around sixty years old, it's actually too much for them, isn't it? Because I come in every day and I tell them: You have to watch out for this. You have to remember that. This is new now. Please pass it on properly. They can't really compensate that. Well, I really pity them, but I have to make sure they can recall it. And this is the worst. I can't be considerate of them because they don't know it. They have to know it just like an 18-year old employee here.	ID 1993<br>Female<br>50 years<br>30 years experience
Changes in social interaction between MAs and patients	Q6	So, everyone in the practice got [vaccine brand]. […] I am glad if I receive a vaccine at all, I also tell that to anyone starting like: "Well yes, but I don't want that one", I tell them: "Be glad if you receive anything at all." Because nobody has ever asked me about the vaccinations against yellow fever, typhus or malaria before their vacation to Africa, nobody has asked me how long these vaccines have been researched, which side effects they have. Never ever.	ID 2322<br>Female<br>58 years<br>41 years experience
Changes in patient numbers	Q7	Afterwards, we had someone with a stroke who didn't say he was already having light dropouts, which would have helped us to get on the right track. We had someone with a heart attack who had typical left-sided chest pain, but he was afraid of going to the hospital and he was afraid of going to the practice […]. It had always been preached [note: by media/government], hadn't it? You should not go unless it's really necessary. And sometimes, people were wondering, well what does really necessary mean.	ID 4579<br>Female<br>49 years<br>28 years experience
	Q8	The wave hits us really hard, and I don't think that many people in the media and in the government are aware that their/ that what they impart just rolls over us like a big wave.	ID 1993<br>Female<br>50 years<br>30 years experience
Patients' anxiety and behavior	Q9	It was really frustrating for me, […] they always think that when consultation hours begin/ well that the girls [note: MAs] are there, period. They don't know that we take care of the urgent ones at noon, we do	ID 4579<br>Female<br>49 years

		the paperwork and then the afternoon begins. 12-hour-shifts are not unlikely to happen, it's pretty easy. And they don't see that, do they?	28 years experience
	Q10	I think it's really bad with younger people, those who could actually get all the information, everything possible, they are usually the ones who/ which doctor do I need to see when I have this referral and so on. And the older ones are reluctant. And those are the ones who don't really know it. There is this difference. Those who could get all the information don't do it, they just want everything perfectly prepared and laid out for them, right? And the older ones don't dare to ask for it. And then I simply ask them. I'm like: "Should I quickly make the call for you? Should I try to find out where to put you?" and so on. Of course, people appreciate that we take a moment and so on, don't they? And I have to say that those who could really need some help don't ask for it a lot. It's usually the others, those who could organize themselves easily, but all of a sudden, they can't do it anymore.	ID 4579 Female 49 years 28 years experience
	Q11	At Christmas for example, there were people who presumably asked why we could not do swabs on the 26th. How this could be possible because when you fly somewhere on the 28th you need one [corona test] that is not older than so and so many hours. This was just one situation where people did not understand that these were holidays like every year and that we were obviously closed, like every year.	ID 4953 Female 39 years 16 years experience
**Superordinate level (politics, legislation, society)**			
Information flow	Q12	You would really like to run a tape: We don't have any more information than the TV gives you. Because, well, unfortunately that's all we know. So, how are we supposed to get more information if the others don't have them either. You know? We don't have any external coming to us who says: This is exactly how it's done. No, we also have to get it from the media somehow.	ID 3332 Female 25 years 2 years experience
	Q13	Well, I think it's really stupid. Yes, it's like, let me put it that way, Mrs. Merkel doesn't govern the country, but individual lands govern themselves and it's as confusing as that for MAs. But, well, ninety or ninety-five percent of the time, you could say that we are on the same path with the exchange. I think you can already notice that everyone thinks it's somehow stupid that it's like that and that there is no general decision for it.	ID 3225 Female 45 years 9 years experience
	Q14	So, eventually it was said that there were corona tests once a week for everyone in [location]. Of course, we do it because we also want to help. We didn't know how to do the billing, we didn't know what to do, we didn't know if we had to type a diagnosis or not. We had to get all the information by ourselves.	ID 7643 Female 36 years

		And I was also sitting there, talking on the phone, doing research on the internet, gathering all the information. So, there was nothing else, nobody told us, this is how you have to do it.	17 years experience
	Q15	And with respect to this test center, the citizens were told: “Yes, you can contact your general practitioner for a transferal.” And neither the city [location] nor the [organization] had any information on how we should transfer the people there. And they were standing at our door then: “Yes, I would like to go to the vaccination center now” And we could only say: “Well, we don’t know how. We don’t have any information.”	ID 3221<br>Female<br>24 years<br>9 years experience
Media reporting	Q16	Well, we are not visible. Just look at the media. Where did we appear? There was a short report on RTL [note: German TV channel] at midnight about all the poor MAs who are at their limits. And that was it. We don’t appear anywhere. We are completely lost. And this always makes me a bit sad. I think: Nobody is interested in us, nobody cared how we were doing, even though we were so close, and we helped to make sure that we take care of the people (as much as we could) as well.	ID 1993<br>Female<br>50 years<br>30 years experience
	Q17	Whenever something is on television or reported in the media. Well, but they were saying this and no, it’s not quite like that, it’s presented differently in the media. Because everyday life looks different, it’s really sugarcoated on television, isn’t it? Not everyone is tested for the mutation, for example. They are only tested if there is concrete evidence or rather if we have a suspicion, like the course of disease has been pretty quick, let’s also test for the mutation.	ID 5573<br>Female<br>39 years<br>19 years experience
Politics and MAs	Q18	Yes, but the point is, where does an ill person go first? Who is contacted first, it’s always the doctor. And it’s not a doctor in the hospital, it’s the GP. And I think we are incredibly disadvantaged or not relevant enough. And at the beginning it was even said that MAs with a positive test are supposed to continue working with FFP2 masks as long as they are not ill, because they are relevant for the system. And yet, the other way round, I hear about this, we clap for retirement homes, we clap for nurses, yes, but you know your people, and we don’t. So, I think it sucks what politicians are doing up there. It’s really – well it doesn’t fit.	ID 2199<br>Female<br>47 years<br>30 years experience
	Q19	And the whole thing was basically happening again, when it was about the orders of vaccination, […] Our turn would have been in summer, everything would have been fine, but suddenly it was said that the teachers, the police officers, all of them should move to the second [prioritization] group. We, in turn, were not supposed to be vaccinated at first, despite having these acute patients every day. In other	ID 4579<br>Female<br>49 years<br>28 years experience

		words, we were put back again, yes, no, people come to us first and another kick in the butt, because you are only vaccinated in the third phase theoretically. […] Our patients actually didn't understand that either, they always thought we had already been vaccinated, didn't they? No, I said, but again, there was some kind of non-appreciation. For me, this was about appreciation.	
	Q20	And I surely didn't receive the test result from one day to the next, but we had to send the educators to one lab and the teachers to a different one – via mail. And we received the results only after five to six days, also by mail. And sometimes you think to yourself: Why am I actually doing this? (laughs). This is of no use to anyone. (laughs) But you're just doing it. Right? Because who am I going to tell that this is nonsense, (laughs) because it doesn't make any sense? If we would have sent it to our lab, we would have done it from one day to the next. That makes sense. It doesn't make sense that you have to wait for it for five days.	ID 1993<br>Female<br>50 years<br>30 years experience
	Q21	Well, people are not aware of the importance of MAs. And politics are definitely not aware of that either. Because else they would [not] cause as much trouble up there, because we are always the fools. Yes, they say: "Go, vaccinate!" "Yes, but how?" None of the politicians has ever taken a syringe and has been through this mess. And that really sucks. This/ they come up with some plans up there and we must execute their ideas! And it's always the same, I'm always thinking, great, nice, thank you. Maybe you should come to the practice and try to implement all that we are supposed to do. I think it's just like that, we are the final link in the chain and yes, we simply have to function then. And if there are any problems, nobody calls any politician to gripe at them, they gripe at us. "Why don't I get my vaccination?" "Yeah, I don't have any. I can't magically summon it."	ID 2199<br>Female<br>47 years<br>30 years experience
Bureaucratic and legal changes	Q22	Or rather, a decision is made, and it has to be implemented tomorrow. It's problematic that there is no lead time to inform everyone, where you can prepare yourselves and read about it before you start. That just doesn't exist.	ID 2046<br>Female<br>32 years<br>11 years since vocational training
	Q23	This was said in early March, that since January there are these regulations and then we had to completely change what we had established two months earlier […] and I […] I really had to check everything again, I had to change diagnoses, I had to change all billing numbers for the whole quarter.	ID 7643<br>Female<br>36 years

		Because for two months, we haven't typed it in correctly and unfortunately, we know the health insurance and they probably won't have mercy on us. And simply delete it, if it's not correct, at least that's what we have assumed now, which is why we wanted to correct it. This made it even harder.	17 years experience
	Q24	Well, unfortunately we mostly had a lot of bureaucratic changes. So that there are different ways of testing and ways of creating corresponding entries in the patient system. And this has changed again and again over the year. At the beginning, it was pretty consistent, then there were returning travelers and you had to use a different form. Now there are citizen tests that can be done for free; all the teacher authorization certificates. So, we thought that everything was quite clear in January and February […] because there weren't many changes, but now in March, a lot has been going on again. And yes, just to mention a few.	ID 3229<br>Female<br>27 years<br>4 years experience
	Q25	In the fourth quarter, there was a whole table on how to do the billing. This overview has changed three times in one quarter. […] So we simply/ We couldn't keep track of it. I had three different sheets with information on how to do the billing in the different periods. And it was basically in the same quarter. It was three months.	ID 2046<br>Female<br>32 years<br>11 years since vocational training
	Q26	A typical day at work is highly affected by bureaucracy and obstacles related to the guidelines. It has been like that for a year now. I have more than 35 years of experience in this job, but I have never experienced something like that before.	ID 2322<br>Female<br>58 years<br>41 years experience
	Q27	Well, yes, we really like to vaccinate, but something's wrong if I have to go through six pages of documentation in advance and copy them and I end up having five folders of documentation for 170 vaccinated people, you know? This can't be right. This can't be the case. It's not about medicine and patient care anymore.	ID 4589<br>Female<br>49 years<br>13 years experience
**Individual level**			
Emotional and psychophysiological reactions	Q28	All of them [the patients] were frustrated, they were scared and so on, but they underestimated one thing and I also said that to one of them afterwards, I said: "You have to think about it. Like I said, we are really trying our best to get you through as quickly as possible, to make sure that you get your things, that everything works, and you are scared, I can accept that, but believe me, every morning when I come	ID 4579<br>Female<br>49 years

		here, I am scared as well", you know? So, that is, we don't have superpowers, not at all, we are also human just like anyone else.	28 years experience
Work-family conflict	Q29	So right now, for example, as a MA you are a bit/ well I have noticed that in my circle of friends or especially with two or three people maybe, that they have kept an extra meter of distance, knowing that I work in a focal practice.	ID 4953<br>Female<br>39 years<br>16 years experience
	Q30	Well, it's quite difficult to find a way there and say: "Okay, now I can go home with a good feeling in the evening, and I can say, I am done for today." Because you often continue and try to get information from other MAs, you browse facebook, you follow all those groups, you try to get as much information as possible and you write everything down in passing. Sometimes, I wrote lists that I presented to my boss the next day, and I was like: "I looked some things up and we could also do this or that."	ID 3221<br>Female<br>24 years<br>9 years experience
	Q31	With small children you always have to be worried about potential closing of daycare centers. Is there an emergency care? Can't we go there? How are we supposed to do that, how to proceed? Is there gonna be another strict lockdown like the last one, where my husband and I had to take care of the children, because daycare centers were indeed completely closed?	ID 6790<br>Female<br>36 years<br>14 years experience
	Q32	Yes, and we also had to fight a lot so that our colleague got her childcare slot at all. We had to update the certificates again and again, with different times, new work times. I also found that too bureaucratic. Well, yeah I would have wished for a facilitation so that you […] wouldn't even be in this situation.	ID 4589<br>Female<br>49 years<br>13 years experience
Job satisfaction and change of job	Q33	Well, I would like to do something else, because it isn't fun anymore, if you just […] Yes, when it comes to appreciation, you know? Well, I have the feeling that people are like/ They think we don't work, you know? That we are just sitting there painting our fingernails and so on. Well, this isn't fun anymore, when you're under so much pressure and you have to live up to what is said in the media, which isn't even true half of the time. This is really annoying.	ID 4205<br>Female<br>44 years<br>28 years experience
